# Frequency of Two Common HFE Gene Mutations (C282Y and H63D) in a Group of Iranian Patients With Cryptogenic Cirrhosis

**DOI:** 10.5812/kowsar.1735143x.781

**Published:** 2011-11-30

**Authors:** Zahra Jowkar, Bita Geramizadeh, Mahmoud Shariat

**Affiliations:** 1 Department of Pathology, Shiraz University of Medical Science, Shiraz, IR Iran; 2 Transplant Research Center, Shiraz University of Medical Science, Shiraz, IR Iran

**Keywords:** Mutation, Iran, Liver Cirrhosis, Genes

## Abstract

**Background:**

The human HFE gene (a key component of iron homeostasis in humans) is involved in hereditary hemochromatosis, a common autosomal recessive genetic disorder that is characterized by excessive intestinal iron absorption and progressive iron overload.

**Objectives:**

In this study, we assessed the frequency of two common forms of hemochromatosis HFE gene mutation (C282Y and H63D) in patients suffering from cryptogenic cirrhosis.

**Patients and Methods:**

One hundred and fifty individuals were included in this study, in which 100 were patients with cryptogenic cirrhosis and 50 were from the normal population. All individuals were examined for common HFE gene mutations by amplification of nucleotide 845 C282Y and 187 H63D alleles and product analysis using the polymerase chain reaction method and restriction enzyme digestion.

**Results:**

No case of either a homozygous or heterozygous C282Y mutation was found. For the H63D mutation, no homozygosity was detected but heterozygosity was detected in 22% of patients and in 28% of the normal population.

**Conclusions:**

Hereditary hemochromatosis is not a major cause of cryptogenic cirrhosis in the Iranian population.

##  1. Background

Hereditary hemochromatosis (HH) is an autosomal recessive condition associated with over-absorption of iron from the intestine and its accumulation in solid organ parenchyma, particularly in the liver, pancreas, and heart [1]. Feder et al. (1996) described a candidate gene on the short arm of chromosome 6, which they named the HFE gene, and identified it as the cause of hemochromatosis [2]. In Western populations, the 2 most common variants detected in this gene, C282Y and H63D, are related to iron overload [3]. Clinical consequences of iron overload include cirrhosis of the liver, cardiac failure, and pancreatic disease [4].

## 2. Objectives

To the best of our knowledge, there are no published studies on the frequency and probable role of HFE mutations in cryptogenic cirrhosis. Therefore, we performed this study to examine the frequency of 2 common mutations of the HFE gene in patients with cryptogenic cirrhosis in the Iranian population.

## 3. Patients and Methods

### 3.1. Patients

In this prospective study, 100 patients with cryptogenic cirrhosis and on a waiting list for a liver transplant were selected. Viral markers and autoimmune markers were negative in these patients. Work up for α-1 antitrypsin deficiency and Wilson's disease were also negative. No history of drug abuse, toxin, or alcoholism was noted. Triglyceride and cholesterol levels were normal in individuals from the control and cirrhotic groups.

Fifty normal, unrelated healthy individuals (49 male and 1 female blood donors) aged 21-61 years (36.98 ± 10) were selected as the control group, all of whom had normal liver function tests. Viral markers were negative in this group. The age of individuals in this group was similar to that of the patient group, but the gender composition of the groups was different because there are very few female blood donors in our region.

### 3.2 Methods

**Figure 1:**
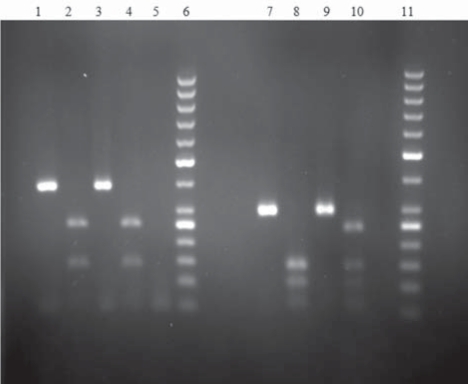
The PCR of the Results of Normal and Heterozygote Patients. Lanes 1, 2, 3, and 4: Normal C282Y before and after enzyme igestion in 2 patients; Lane 5: Negative Control; Lane 6: 0-bp ladder; Lanes 7 and 8: H63D before and after enzyme igestion in a normal patient; Lanes 9 and 10: H63D efore and after enzyme digestion in a heterozygote patient; ane 11: 50-bp ladder.

A 10-mL sample of fasting blood was collected from each patient for HFE gene mutation analysis, biochemical analysis, and to measure transferrin saturation (TS). DNA was extracted from blood leukocytes using a DNA isolation kit (Qiagen Company , USA). Polymerase chain reaction-restriction fragment length polymorphism (PCR-RFLP) analysis was then performed(Figure 1).

## 4. Results

Among the 100 patients with cryptogenic cirrhosis that were examined, we found no homozygotes or heterozygotes for C282Y. Although no homozygote case was detected for H63D, 22 of the patients (22%) were heterozygous for H63D. In the control group, 14 patients (28%) were heterozygous for H63D but no homozygote cases were detected. Further, no C282Y mutation, either homozygous or heterozygous, was found.Table 1 shows the HFE gene mutation status of the two control groups and of cirrhotic patients in the iron-overloaded and normal groups. As indicated in Table 1, most of the cirrhotic patients and controls with heterozygous mutations had a normal iron load.

**Table 1:** The Frequency of HFE Gene Mutations in Two Groups of Cirrhotic Patients and Normal Blood Donors, According to the Presence or Absence of ron Overload

	** C 282Y, No.**(%)		** H63D, NO. **(%)		** No Mutation, No. **(%)
	**Homozygote**	**Heterozygote**	**Homozygote**	**Heterozygote**	
Patients (n = 100)					
Iron overload	0	0	0	1 (1)	13 (13)
Normal iron	0	0	0	21 (21)	65 (65)
Normal controls (n = 50)					
Iron overload	0	0	0	1 (2)	2 (4)
Normal iron	0	0	0	12 (26)	34 (68)

## 5. Discussion

Hereditary Hemochromatosis (HH) is a genetic disorder, the prevalence of which varies in different ethnic groups. The expression of HH is modified by several factors, including dietary iron intake and blood loss associated with menstruation, pregnancy, and blood donation [5]. The gene responsible in the majority of patients with this disorder (the HFE gene) has been identified on the short arm of chromosome 6, and encodes a 343-amino acid protein [6]. Thirty-seven allelic variants of HFE gene mutations have been identified. Two of the missense mutations that are found in the majority of patients with HH are C282Y and H63D [3]. The frequency of the most common HFE genotype differs in different geographic locations [7]. In this study, we investigated the probable association of HFE gene mutations in a group of patients with cryptogenic cirrhosis. As shown inTable 2, no homozygous cases of either C282Y or H63D were detected and only 22% of the patients were heterozygous for H63D, which was not significantly different from the controls (28%) (P > 0.05).

To the best of our knowledge, few studies have examined the frequency of HFE gene mutations in patients with chronic liver diseases and cirrhosis [8]. However, as indicated in Table 2, our results are very similar to those reported in India and relatively similar to those reported in Turkey [1][3]. Previous studies have described the prevalence of HFE gene mutations in the Iranian population, in patients with thalassemia, and in a group of diabetics [8][9][10]. The results of both these studies were very similar to ours with regards to H63D. However, we did not observe any cases of heterozygotes for C282Y, whereas the frequency of heterozygotes in patients with thalassemia was 3% in a previous study from Iran [8][9][10][11].

**table 2:** Comparison of the Previous Studies Regarding the Frequency of HFE Gene Mutations Among Patients With Different Types of Chronic Liver iseases in Different Geographic Locations

	**Target of Study**	**C282Y, %**		**H63D, %**	
		**Heterozygote**	**Homozygote**	**Heterozygote**	**Homozygote**
USA[2]	Cryptogenic cirrhosis	5.4	0.26	13.5	1.89
USA[12]	HCV cirrhosis	11.5	0	0	0
Western Romania[13]	Various liver diseases	4.8	19	9.5	0
Germany [14]	Chronic hepatitis C	6.6	0	34.9	3
Brazil [15]	Chronic hepatitis C	35	0	5	0
Turkey [16]	Chronic liver disease	1.7	0	28	2
India [17]	Chronic liver disease	0	0	14.8	0.4
India [3]	Cryptogenic cirrhosis	0	0	19.3	3.2
Iran (Current study)	Cryptogenic cirrhosis	0	0	22	0

In conclusion, our study suggests that iron overload and HFE gene mutations do not play a primary role in cryptogenic cirrhosis in the south Iranian population.
